# Is coracoclavicular reconstruction necessary in hook plate fixation for acute unstable acromioclavicular dislocation?

**DOI:** 10.1186/s12891-021-03978-3

**Published:** 2021-02-01

**Authors:** Yu-Ta Chen, Kuan-Ting Wu, Shun-Wun Jhan, Shan-Ling Hsu, Hao-Chen Liu, Ching-Jen Wang, Jih-Yang Ko, Wen-Yi Chou

**Affiliations:** Department of Orthopedic Surgery, Kaohsiung Chang Gung Memorial Hospital, 123 Ta Pei Road, Niao Sung Dist, Kaohsiung, Taiwan

**Keywords:** Acromioclavicular joint dislocation, Hook plate, Coracoclavicular reconstruction, Loop suspensory reconstruction

## Abstract

**Background:**

Acromioclavicular joint (ACJ) dislocation is a relatively common shoulder injury. For the treatment of cases of severe ACJ dislocation (Rockwood type III–V), hook plate fixation is an easy-to-master and minimally-invasive approach to surgical intervention. Over stress on the acromion following hook plate fixation often leads to acromial complications such as osteolysis and loss of reduction. We hypothesized that suspensory reconstruction alongside hook plate fixation might provide a superior stability and reduce complications as compared with hook plate fixation alone. The purpose of the study was to assess the clinical and radiographic outcomes of these two surgical modalities.

**Methods:**

We retrospectively enrolled 49 patients with acute ACJ dislocation from May 2010 to December 2018. Among them, 19 patients received hook plate fixation only (HP group), and 19 underwent concomitant hook plate fixation and loop suspension fixation with two mersilene sutures (HM group). The demographic data of the patients were recorded and analyzed. All patients underwent a shoulder X-ray initially, immediately postoperatively, and at 1, 3, 6 and 12 months to measure the relative coracoclavicular distance (rCCD). Clinical assessment of shoulder function outcome was conducted using the Constant Murley Score (CMS); the University of California at Los Angeles (UCLA) Shoulder Score was also measured at the latest follow-up.

**Results:**

There were no significant differences in the demographic data between the two groups. With regards to the CMS and the UCLA score, the HM group and HP group both had excellent outcomes, and no significant differences in scores were observed between groups (CMS: 93.90 ± 6.16 versus 94.47 ± 7.26, *p* = 0.47; UCLA score: 32.84 ± 2.91 versus 34.32 ± 1.16, *p* = 0.07). However, the HM group demonstrated substantial superiority in terms of maintenance of the rCCD over the HP group (91.47 ± 27.47 versus 100.75 ± 48.70, *p* = 0.015). In addition, there was less subacromial osteolysis in the HM group than the HP group (52.6% versus 15.8%, *p* = 0.038).

**Conclusion:**

Both fixations yielded excellent functional outcomes. However, concomitant hook plate fixation with loop suspensory reconstruction demonstrated the fewer acromion complications and statistical differences in reduction maintenance with less clinical significance.

## Background

Acromioclavicular joint (ACJ) dislocation is a relatively common shoulder injury in active young males [1, 2], usually sustained during a fall or in contact sports with direct force to the acromion under an adducted arm [3]. The ACJ is an important structure connecting the axial skeleton and upper extremities, the upper extremities being suspended by a strong coracoclavicular (CC) ligament and an acromioclavicular (AC) ligament. Thus, ACJ dislocation with torn AC and CC ligaments often leads to severe functional impairment of the injured shoulder. Appropriate treatment is necessary in the acute phase of ACJ owing to the healing potential of the CC ligament [4]. Different treatment strategies have been proposed according to the severity of ACJ dislocation, which is classified based on the magnitude and direction of dislocation. Low-grade injuries, such as Rockwood type I and II ACJ dislocations, respond well to conservative treatment. However, high-grade dislocations (Rockwood type IIIB, IV, V, VI) still remain controversial. Though some author favor nonoperative management at first [5, 6], the aggressive surgical intervention is usually recommended by the literature review [3, 7, 8]. Surgical fixation has been advised for the acute high-grade ACJ dislocation based on superior healing potential of the CC ligament after reconstruction [4]. Otherwise, biologic ligament reconstruction should be taken into consideration in patients with chronic ACJ instability [6, 9].

Numerous surgical modalities have been proposed for high-grade ACJ dislocations, which can be divided into either AC-stabilizing or CC-stabilizing techniques. AC-stabilizing techniques include intra-articular fixation, such as with Kirschner wires, threaded pins, and hook plate fixation, etc., while CC-stabilizing techniques or extra-articular fixation can be accomplished with coracoacromial ligament transfer (Weaver–Dunn procedure), ligament reconstruction, suture anchor, or an endo-button device. To date, the optimal surgical technique for ACJ dislocation is still under debate owing to controversy in reported outcomes. In a recent study, hook plate fixation was reported to be a popular option that provides rigid fixation and promotes nature scaring of the CC ligament, with the advantages of a simpler surgical technique, minimally-invasive access, and early resumption of normal activity [4, 10–12]. Despite these advantages, hook plate fixation also has disadvantages, which include the need for implant removal surgery, subacromial impingement, subacromial osteolysis, and possible loss of reduction after implant removal, which may lead to complications such as a rotator cuff tear or an acromion fracture in patients with osteoporosis or those with a high activity level [13–15].

The hook plate serves as a secure fixation device, with a hook that transfers superior migration stress from the distal clavicle to the undersurface of the acromion. However, the persistent high pressure often leads to subacromial osteolysis [15] and patient with delay scaring of CC ligament may loss of reduction after removed of hook plate. Therefore, we hypothesized that concomitant hook plate fixation with loop suspension reconstruction would yield better stabilization, with a lower acromial loading that results in superior clinical outcomes and fewer complications. In the present study, we aimed to compare the functional and radiographic results in patients with high-grade ACJ dislocations treated with hook plate fixation alone or concomitant hook plate fixation with CC suspension reconstruction.

## Methods

### Patient enrollment

**Fig. 1:**
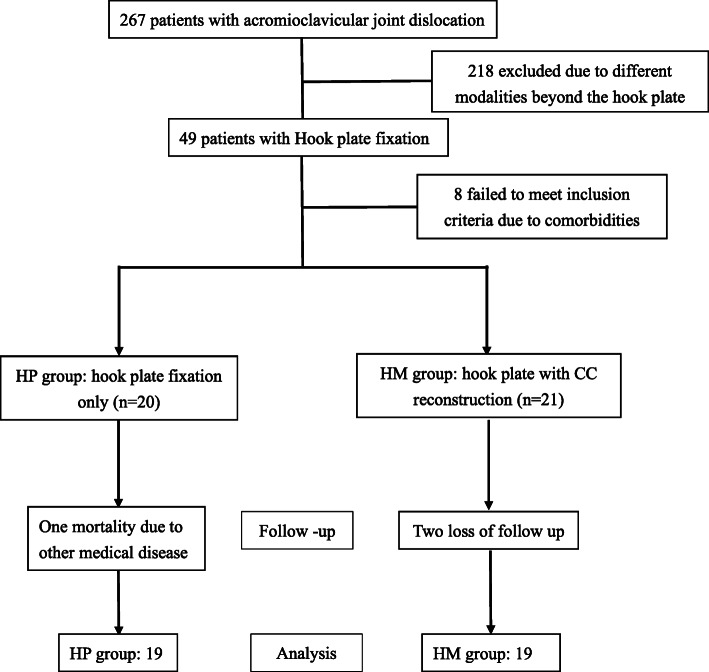
Flowchart of patient recruitment to the HP group and the HM group. HP: hook plate; HM: hook plate with mersilene suture reconstruction

This retrospective comparative study was conducted following receipt of approval from our institutional review board. The inclusion criteria were as follows: 1. age > 18 years; 2. unilateral injury; 3. acute injury(< 4 weeks); and 4. high-grade ACJ dislocation (Rockwood type III–VI). Patients with the following conditions were excluded: additional fractures (clavicle, scapulae, or proximal humerus) in the same shoulder, ACJ arthritis, or rotator cuff injury. Patients with a previous injury to the same shoulder and those who were followed-up for less than 1 year were also excluded from the study. From May 2010 to December 2018, 267 patients with high-grade ACJ dislocations (Rockwood classification type III–V) underwent surgical interventions. The existence of controversy from surgeon to surgeon, the modalities could be divided into CC reconstruction with horizontal K wires fixation and hook plate fixation. Forty-nine of the 267 patients were treated using hook plate fixation (DePuy Synthes 3.5 mm LCP® Clavicle Hook Plate or Aplus® Distal Clavicle HOOK Locking Plate System) and another 218 patients treated with intra-articular Kirschner wires fixation, loop suspension fixation or biologic ligament reconstruction. There were totally six surgeons applied the hook plate fixation in the beginning and the additional CC reconstruction with non-absorbable, braided, sterile polyester surgical suture (Mersilene® Polyester Fiber Suture, Ethicon, Cincinnati, OH, USA) were developed later for the enforcement of vertical soft tissue stability instead of hook plate only (Fig. 1).

Finally, 38 patients were recruited in this study. Nineteen patients underwent hook plate fixation alone (HP group), and the other 19 patients underwent hook plate fixation with CC reconstruction using mersilene suture (HM group). Two patients were injured due to falling from a standing height, and the others were involved in motorcycle accidents. All hook plates were removed 3 to 6 months after the index surgery with retained mersilene suture. For the symptomatic subacromial osteolysis, the hook plates will be removed 3 months after the index surgery. Otherwise, the implants retained until 6 months.

### Pre-and postoperative assessment

Demographic and clinical data were recorded, including age, gender, mechanism of injury, Rockwood classification, interval between injury and surgery, and timing of implant removal. Shoulder functional assessment was conducted using the University of California at Los Angeles (UCLA) Shoulder Score [16], the Constant Murley Score (CMS) [17] and ACJ-specific Taft score [18], which includes subscales to assess pain (0–10), night pain (0–5), strength (0–25), activities of daily living (0–20) and range of motion (0–40) and Taft score is the sum of subjective evaluation (pain and stiffness), objective evaluation (ROM and strength compare with opposite shoulder) and radiographic examination (AC joint subluxation, dislocation or post-traumatic osteoarthritis). The subjective pain score was measured using a visual analog scale (VAS). All clinical evaluations were carried out at 1, 3, 6 and 12 months postoperatively.

### Surgical intervention

**Fig. 2:**
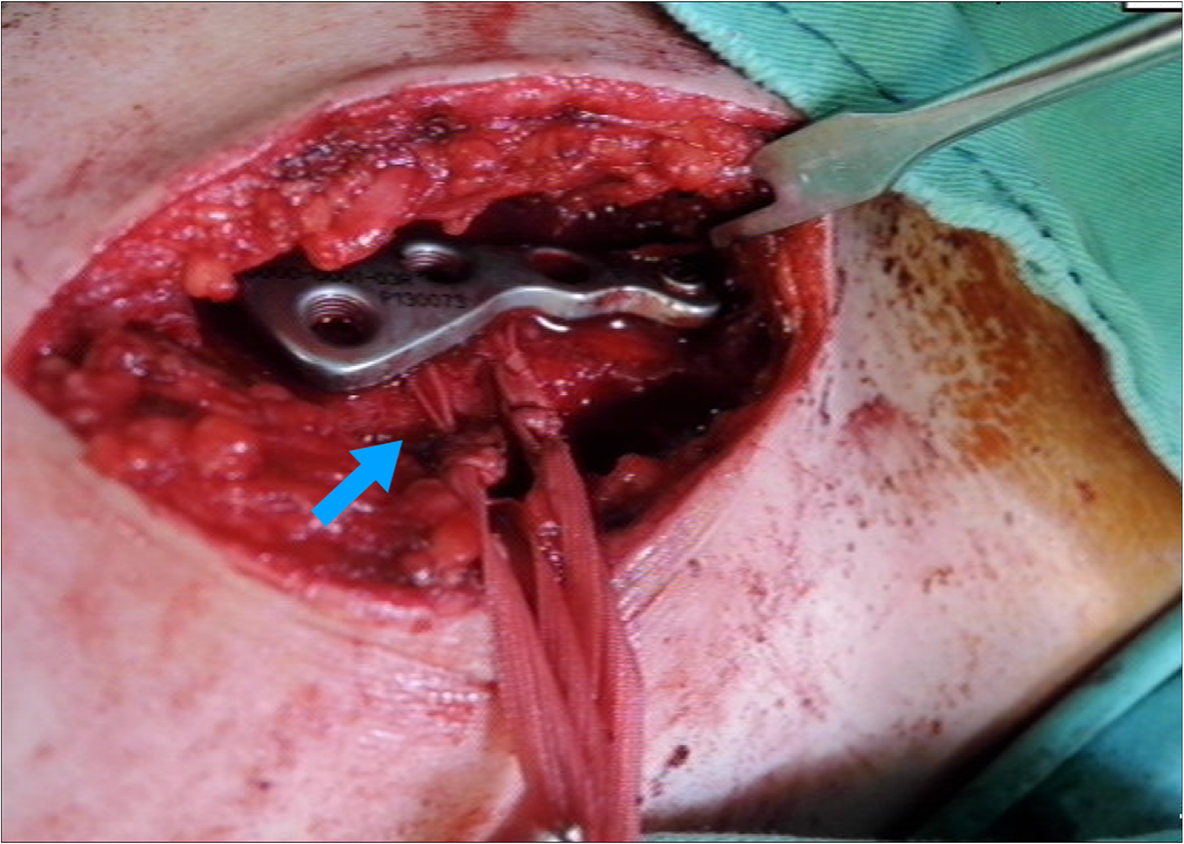
Intra-operative illustration of Hook plate fixation with two Mersilene suture reconstruction. This case was suffered from right shoulder high grade ACJ dislocation after traffic accident. In operation, two Mersilene suture had been suspended at first, then Aplus hook plate had been applied. Blue arrow means two bundle of Mersilene suture reconstruction

Patients were placed in a beach-chair position under general anesthesia. The approach began from the AC joint at the anterior one-third of the distal clavicle with a 5–6-cm transverse incision, then the ruptured meniscus and hematoma in the ACJ were debrided. The ACJ was reduced and provisionally fixed using k-wire. In the HP group, an appropriate clavicular hook plate was inserted directly posterior to the ACJ, with the hook portion under the acromion, and the clavicle was part-fixed with screws. In the HM group, CC reconstruction was performed at the beginning by passing two mersilene sutures just underneath the coracoid process with right-angle dissectors; then, two clavicle tunnels of a 2.7 mm width were created 3–4-cm medial to the distal clavicle end between the trapezoid and conoid ligament. The passed mersilene sutures were tied through the clavicle bone tunnels under slight over-reduction of the ACJ. Then, the hook plate applied with the sparing of the clavicle tunnel from screw insertion (Fig. 2). Finally, the ACJ capsule and deltotrapezial fascia were repaired using absorbable sutures. All patients tolerated the procedure well, there were no major complications such as neurologic or vascular injury.

### Radiographic assessment

**Fig. 3:**
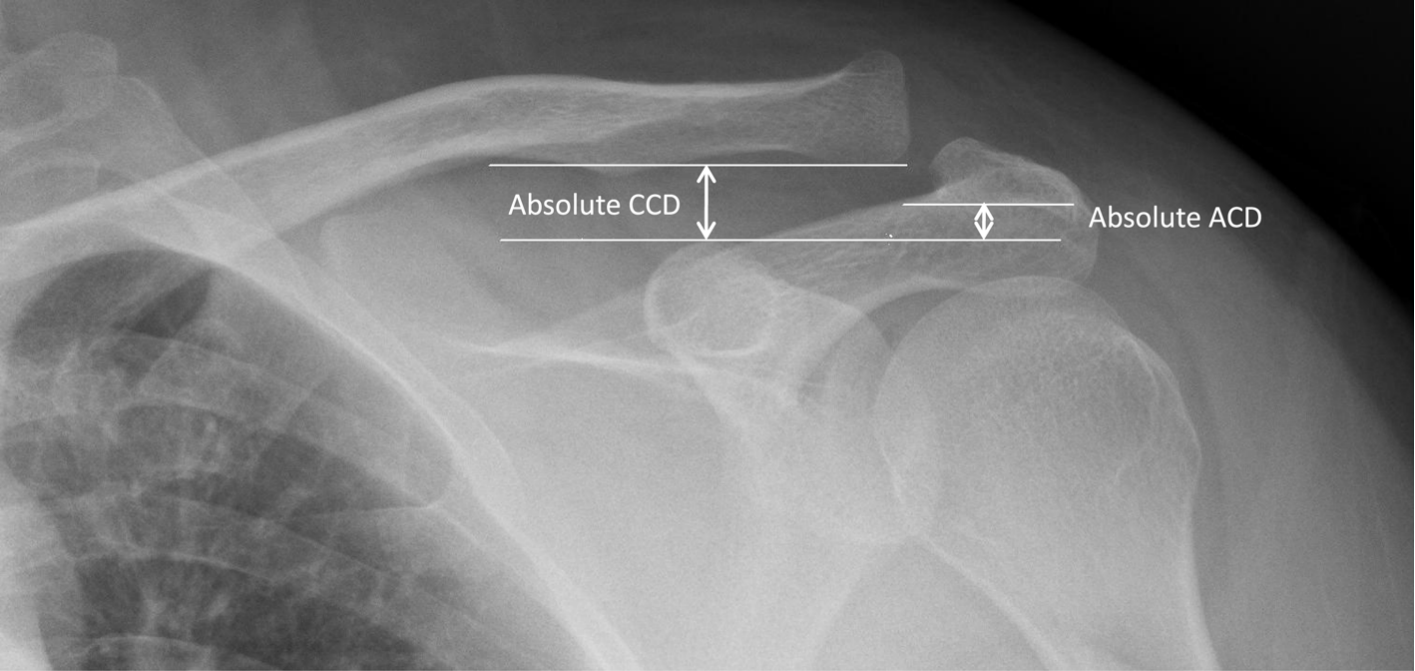
Relative CCD = absolute CCD* 100% / absolute ACD. ACD, acromioclavicular distance; CCD, coracoclavicular distance

A series of plain films, including AP and outlet views, was obtained prior to surgery, on postoperative day 1, and 1, 3, 6 and 12 months postoperatively. In the radiographic assessment, three lines were drawn horizontal to the ground: the coracoidal parallel line was drawn through the superior cortex of the coracoid; the acromial parallel line was drawn through the inferior acromial cortex; and the clavicular parallel line was drawn through the inferior clavicular cortex [19]. The absolute coracoclavicular distance (aCCD) refers to the distance between the clavicular parallel line and the coracoid parallel line, while the absolute acromiocoracoid distance (ACD) was defined as the distance between the acromial parallel line and the coracoidal parallel line. The relative coracoclavicular distance (rCCD) was defined as the ratio of the aCCD to the ACD (aCCD/ACD*100%) (Fig. 3). Subacromial osteolysis refers to radiolucent signs around the hook and subacromial space. Distal clavicle osteolysis assessed after remove hook plate. All radiographic examination measurement conducted by a single orthopedic surgeon who did not participate in the surgeries.

### Rehabilitation

The shoulders operated upon were protected by the use of a shoulder sling for 6 weeks. Passive exercise was initiated immediately after surgery via low-grade forward flexion and pendulum exercises. Active and rotational motion was carried out 4 weeks postoperatively, and muscle strengthening was initiated after 6 weeks under tolerable pain. Full ROM was permitted before removal of the hook plate. All patients received the similar protocol in our institute, but 1–2 weeks variations existed due to individual difference of recovery.

### Statistical analysis

Continuous variables are expressed as the mean with one standard deviation unless otherwise specified. Categorical variables were evaluated using the Fisher exact test for nonparametric statistics due to the small sample size. The two-tailed Mann-Whitney U test was used for all continuous variables. The significance level was set at 0.05 (*p* < 0.05). Data were analyzed using SPSS 22.0 for Windows (SPSS, Inc., Chicago, IL, USA).

## Results

### Patient demographics

A total of 38 patients with acute ACJ dislocation who underwent hook plate fixation were included in this study, 19 patients in the HP group, and 19 in the HM group, with a mean age of 44.47 ± 15.41 and 46.42 ± 16.41 years (*p* = 0.73), respectively. There were no statistically significant differences in patient demographics, including gender, age, and injury site and severity, between the two groups. However, male patients were predominant in both groups, and nearly all patients were victims of motorcycle accidents. The time to surgery in the HM group was longer than that in the HP group (5.47 ± 5.90 days versus 2.16 ± 1.64 days, respectively, *p* = 0.271), without statistical significance. Regarding implant removal, the hook plate was removed at 5.32 ± 1.46 months in the HP group and 5.68 ± 1.63 months in the HM group (*p* = 0.385) (Table 1).

**Table 1:** Patient demographic data

	HP(19)	HM(19)	*P* value
Age	44.47 ± 15.41	46.42 ± 16.41	0.73
Gender			0.714
Male	15	13	
Female	4	6	
Site			0.313
Left	10	14	
Right	9	5	
Rockwood classification			1.00
III	9	10	
V	10	9	
Time to surgery (days)	2.16 ± 1.64	5.47 ± 5.90	0.271
Plating time (Months)	5.32 ± 1.46	5.68 ± 1.63	0.385
Follow time after remove hook plate (Months)	32.47 ± 24.85	26.42 ± 22.48	0.096
Follow up (Months)	38.53 ± 24.90	32.68 ± 21.73	0.172

### Functional outcome

The mean follow-up duration was 38.53 ± 24.90 months in the HP group and 32.68 ± 21.73 months in the HM group (*p* = 0.172). There was no significant difference in the overall CMS between the two groups, at 94.47 ± 7.26 and 93.90 ± 6.16 (*p* = 0.47), respectively. Similar results were obtained in subgroups in terms of the CMS (Pain: 14.84 ± 0.37 vs. 14.74 ± 0.56, *p* = 0.75; Activity and daily: 9.90 ± 0.32 vs. 9.68 ± 0.75, *p* = 0.56; ROM: 38.63 ± 2.01 vs. 37.90 ± 3.43, *p* = 0.82; Strength: 21.05 ± 5.85 vs. 21.97 ± 3.29, *p* = 0.89) and the UCLA score (34.32 ± 1.16 vs. 32.84 ± 2.91, *p* = 0.07). There was no significant difference in the VAS overall pain score between groups (1.17 ± 0.38 vs. 1.19 ± 0.54, *p* = 0.75) (Table 2).

**Table 2:** Functional outcome

	HP(19)	HM(19)	*P* value
VAS	1.17 ± 0.38	1.19 ± 0.54	0.75
CMS	94.47 ± 7.26	93.90 ± 6.16	0.47
Pain	14.84 ± 0.37	14.74 ± 0.56	0.75
Activity and daily	9.90 ± 0.32	9.68 ± 0.75	0.56
ROM	38.63 ± 2.01	37.90 ± 3.43	0.82
Strength	21.05 ± 5.85	21.97 ± 3.29	0.89
UCLA	34.32 ± 1.16	32.84 ± 2.91	0.07
Taft score	10.21 ± 1.57	10.61 ± 1.32	0.62

### Radiographic outcome

**Fig. 4:**
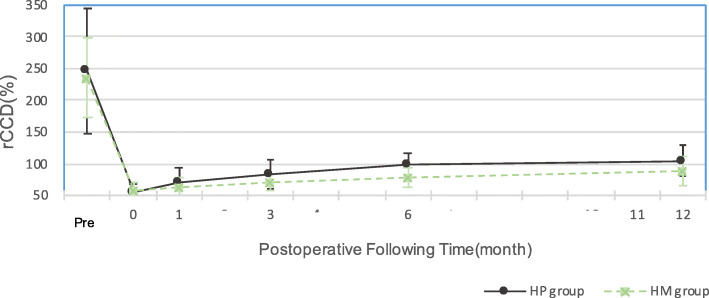
Radiographic outcomes of the HP and HM groups. The line chart illustrated the preoperative relative CC distance and the trend of rCCD of the HP and HM groups by postoperative follow-up time

**Fig. 5:**
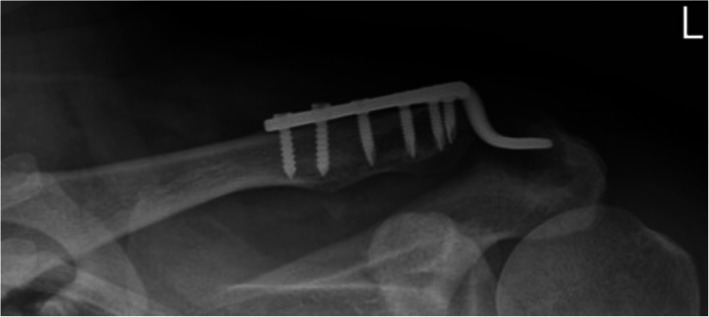
Severe subacromial osteolysis after Hook plate fixation with loss of reduction. This patient with left shoulder ACJ dislocation, status post hook plate fixation only for 6 months. Before remove Hook plate, left ACJ loss of reduction was noted with severe acromion osteolysis

As shown in Table 3, the preoperative relative CC distance (rCCD) was 247.31 ± 98.05% in the HP group and 234.60 ± 62.11% in the HM group (*p* = 0.795). Both groups revealed significant improvement in the rCCD (*p* < 0.001) after surgery, without significant difference between groups (HP vs HM = 56.34 ± 12.82 vs. 57.99 ± 12.21, *p* = 0.773). During follow-up, mild progressive loss of reduction was observed from postoperative month 1 until month 12, and the difference in the rCCD in both groups became statistically significant from month 3 to month 12 postoperatively (Fig. 4). Besides, the rCCD at 12 months still exhibited significant improvement as compared with the preoperative rCCD (*p* < 0.001, both groups). Delta rCCD was defined as the increased amount of postoperative month rCCD compared with postoperative immediately. Cohen’s d in each month had been calculated, from 0.6 (postoperative 1 month) to 1.2 (postoperative 6 month), and finally are 0.3 (postoperative 12 months). Subacromial osteolysis after plate removal was observed in both groups, affecting 10 patients in the HP group, but only three in the HM group (*p* = 0.038) (Fig. 5). No infection case was noted in both groups. Two cases in HP group and one case in HM group had distal clavicle osteolysis.

**Table 3:** Radiographic outcome

	HP(19)	HM(19)	*P* value
Preop rCCD			
Absolute ACD (mm)	11.57 ± 5.21	10.33 ± 2.86	
Absolute CCD (mm)	24.63 ± 4.97	22.96 ± 4.14	
Relative CCD(%)	247.31 ± 98.05	234.60 ± 62.11	0.795
Postop rCCD			
Absolute ACD (mm)	19.41 ± 9.00	18.40 ± 9.82	
Absolute CCD (mm)	11.20 ± 6.63	11.10 ± 7.37	
Relative CCD(%)	56.34 ± 12.82	57.99 ± 12.21	0.773
*p* value (postop-preop)	< 0.001	< 0.001	
Correct rCCD(%)	190.97 ± 98.9	176.61 ± 64.58	0.885
Postop 1 month rCCD			
Absolute ACD (mm)	17.73 ± 9.03	12.64 ± 6.50	
Absolute CCD (mm)	12.32 ± 6.96	7.91 ± 4.98	
Relative CCD(%)	70.80 ± 21.80	62.66 ± 15.66	0.212
Postop 3 months rCCD			
Absolute ACD (mm)	15.93 ± 9.43	13.38 ± 6.00	
Absolute CCD (mm)	12.67 ± 7.41	9.19 ± 4.32	
Relative CCD(%)	82.96 ± 22.57	69.80 ± 13.26	0.050*
Postop 6 months rCCD			
Absolute ACD (mm)	14.37 ± 8.01	14.48 ± 5.76	
Absolute CCD (mm)	13.33 ± 6.59	11.63 ± 5.45	
Relative CCD(%)	97.59 ± 19.87	79.29 ± 15.51	0.004*
Postop 1 year rCCD			
Absolute ACD (mm)	13.24 ± 8.80	12.20 ± 4.31	
Absolute CCD (mm)	14.36 ± 7.91	10.6 ± 2.70	
Relative CCD(%)	100.75 ± 48.70	91.47 ± 27.47	0.015*
∆rCCD			
∆ rCCD(1 M)	14.46 ± 20.16	4.67 ± 10.92	0.172
∆ rCCD(3 M)	26.62 ± 23.21	11.8 ± 11.4	0.053
∆ rCCD(6 M)	41.24 ± 20.90	21.30 ± 11.11	0.006*
∆rCCD(1Y)	45.76 ± 50.43	31.14 ± 19.36	0.034*
*p* value (Postop 1y-preop)	< 0.001	< 0.001	
Complication			
Infection	0	0	1
Acromion osteolysis	10	3	0.038*
Distal clavicle osteolysis	2	1	0.547

*Statistically significant; **∆**rCCD means postop (month) rCCD minus postop rCCD

## Discussion

The principal finding of the present comparative study was that concomitant CC reconstruction with hook plate fixation provided the statistical differences in reduction maintenance and reduced the incidence of acromial osteolysis as compared with hook plate fixation alone in acute high-grade ACJ dislocations, although there was no significant difference in the functional outcome. In recent decades, hook plate fixation has become a popular option owing to the lesser requirement for dissection and simple application, allowing early shoulder girdle exercise, and with probably the same or a lower complication rate as compared with conventional pinning techniques [11, 12].

Several studies have reported satisfactory functional outcomes of hook plate fixation. Stein et al. prospectively recruited 27 high-grade (Rockwood grade IV/V) ACJ dislocation patients who underwent hook plate fixation, and after a 24-month follow-up period, the patients exhibited a good to excellent functional outcome (Constant score: 90.19 ± 7.79) [19]. Arirachakaran et al. pooled 11 studies of patients undergoing hook plate fixation in a systemic review, and also disclosed excellent functional outcomes (Constant score: 90.35 ± 3.19) [11]. Huang et al. treated 24 acute-type V AC joint dislocations with hook plate fixation; all patients had satisfactory outcomes (UCLA score: 33.0 (29–35)), and the rCCD was better in that group than in the mersilene suture group after an one-year follow-up period [12]. In this study, we also demonstrated significant improvements in functional outcome (94.47 ± 7.26 and 93.90 ± 6.167) and the rCCD (HP:247.31 ± 98.05% to 56.34 ± 12.82%, *p* < 0.001; HM:234.60 ± 62.11% to 57.99 ± 12.21%, *p* < 0.001) in both the HP and HM groups.

Several studies have compared the clinical outcomes between loop suspension reconstruction and hook plate fixation, and reported superior outcomes in the loop suspension groups. In a meta-analysis, Arirachakaran et al. revealed that loop suspension fixation resulted in a higher functional outcome than hook plate fixation but no significant (Constant score: 92.84 ± 1.57 versus 90.35 ± 3.19, 95% confident interval from − 1.43 to 5.69) [11], while Stein et al. also disclosed a more favorable outcome of loop suspension as compared with hook plate 272 fixation (Constant score: 95.3 ± 4.4 versus 90.2 ± 7.8, *p* = 0.02) [19]. In a comparison of tight rope fixation and hook plate fixation, Bin Abd Razak HR et al. reported a better CMS in the tightrope group (87.6 ± 11.7 versus 77.5 ± 12.3, *p* = 0.046) [20]. The inferior functional outcome of hook plate fixation may be attributed to different rehabilitation protocols, concomitant lesions, and vertical or horizontal instability after removal of the implants [19]. Controversial existed in the direct comparison of CC reconstruction versus hook plate fixation in literature review [11–13, 19, 21]. We supposed the combined procedures would offer the better functional and radiographic outcome other than a single procedure although time consuming. Our analysis demonstrated a lower rCCD in the HM group than in the HP group since 3 months postoperatively (69.80% ± 13.26% versus 82.96% ± 22.57%, *p* = 0.05) and a significantly lower rCCD at the postoperative one-year follow-up (91.47 ± 27.47 versus 100.75 ± 48.70, *p* = 0.015). Concerning the effect size is small, we concluded that CC reconstruction in hook plate fixation offered the statistical significance in rCCD maintenance and reduction of acromial osteolysis. Therefore, CC reconstruction in hook plate fixation could offer the superior radiographic outcomes in CC distance maintenance and reduction of subacromial osteolysis. Therefore, we presumed that concomitant CC reconstruction with hook plate fixation could reduce the vertical instability with load-sharing from the acromion to the coracoid and clavicle, especially after implant removal. In this study, we demonstrated a lower rCCD in the HM group than in the HP group from 3 months postoperatively (69.80% ± 13.26% versus 82.96% ± 22.57%, *p* = 0.05) and a significantly lower rCCD at the postoperative one-year follow-up (91.47 ± 27.47 versus 100.75 ± 48.70, *p* = 0.015).

**Fig. 6:**
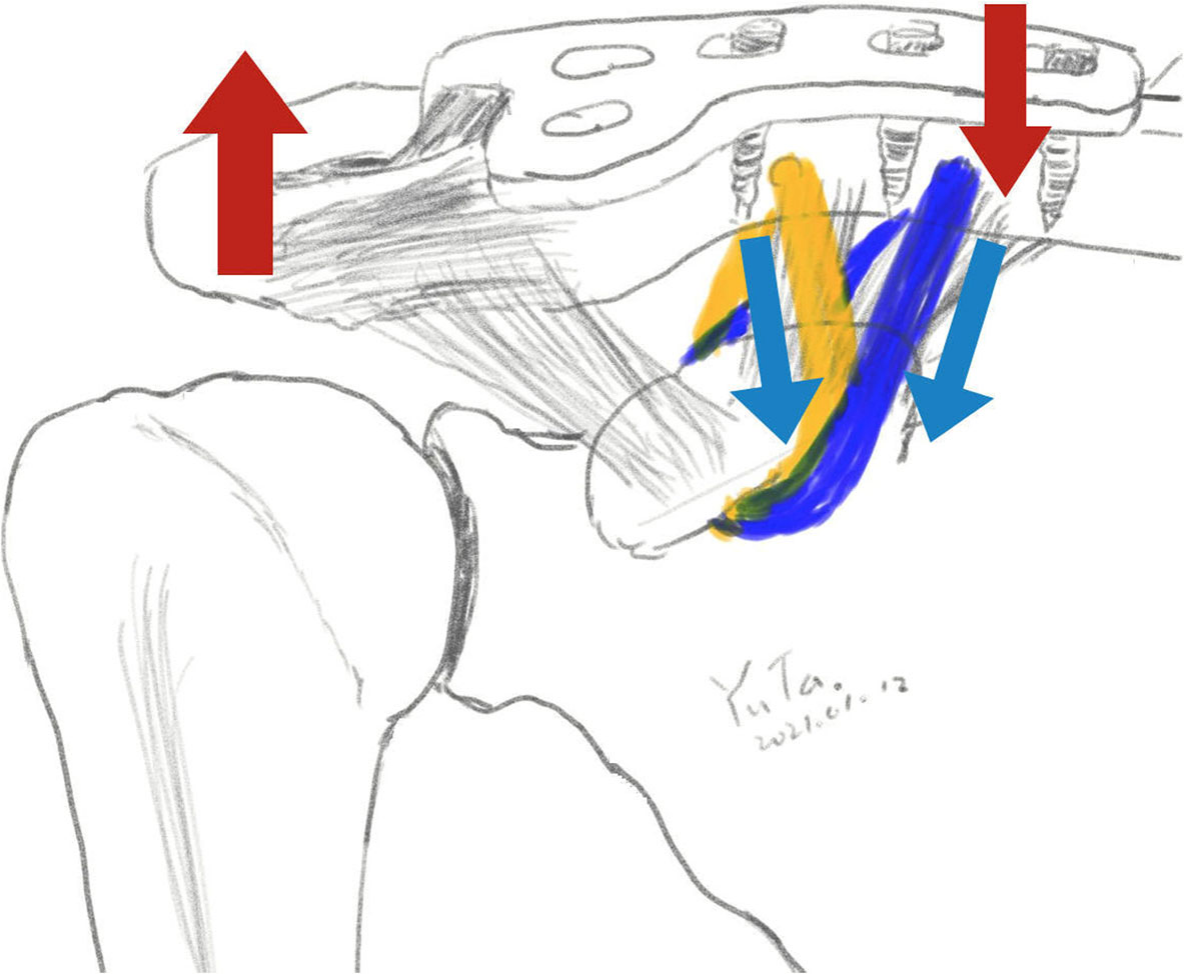
Mechanism of Mersilene suture alleviated hook pressure of hook plate. Hook of hook plate will provide strongly vertical stability by attached to inferior cortex of acromion. However, in hook plate fixation without Mersilene suture augmentation, high pressure of hook tip will cause subacromial osteolysis and possible loss of reduction of AC joint (Red arrow). With Mersilene suture augmentation, pressure of hook tip will be alleviated and decreased possible of subacromial osteolysis and provide vertical stability after remove hook plate (Blue arrow)

With coracoclavicular reconstruction, the vertical force on the ACJ is shared, which alleviates pressure over the hook before implant removal and maintains the rCCD subsequently (Fig. 6). In a case–control study by Wang et al., there were fewer cases of recurrent AC instability in patients who underwent hook plate fixation combined with acromiocoracoid ligament transfer than in those who underwent hook plate fixation alone [22]. The augmentation of mersilene suture with hook plate fixation in one stage resulted in a better rCCD and a lower incidence of subacromial osteolysis owing to pressure alleviation over the hook of the hook plate and maintenance of vertical stability after removal of the hook plate. Yin et al. reported a similar outcome following study of the use of a hook plate with or without double-tunnel coracoclavicular ligament reconstruction. In the hook plate fixation alone group, six patients had loss of reduction (23.08%), and 12 patients had acromion cortex erosion, but no related complications were observed in the ligament reconstruction group [21]. In this study, we observed a similar CMS in the HM and HP groups (94.0 ± 6.54 versus 94.2 ± 7.35, *p* = 0.75); however, the grade of loss of reduction was better in the HM group 12 months after surgery (100.75 ± 48.70 versus 91.47 ± 27.47, *p* = 0.015), indicating that the HM group exhibited the statistical differences in reduction maintenance over the HP group.

Regarding hook plate fixation, the hook plays an important role in stabilization in ACJ dislocation, but the focused high pressure over the hook tip may cause erosion of bone cortex (Fig. 6). Among patients with hook plate fixation, 25–50% suffer subacromial osteolysis or erosion [13, 23–25], which are the most common complications in hook plate fixation. Subacromial osteolysis may result in more postoperative pain, discomfort, and an impaired functional outcome [10, 13]. Yoon et al. also reported a trend of an inferior functional score in patients with subacromial osteolysis [13], which indicated that greater stress on the hook tip may lead to a greater risk of subacromial osteolysis. In the present study, the incidence of acromial osteolysis was lower in the HM group than the HP group (52.6% versus 15.8%, *p* = 0.038), meaning that CC reconstruction exerted a load-sharing effect on the acromion.

Despite the promising results of this study, there were limitations that should be addressed. First, this was a retrospective, non-randomized control study, suggesting that bias may exist regarding the homogeneity of the hook plate group and the loop suspension fixation group. Second, the limited sample size and relatively short follow-up duration might weaken the strength of the results. Third, the advanced assessment for the ACJ disorders did not perform due to insufficiency of clinical significance from VAS, UCLA score, CMS and Taft score. Finally, strict biomechanical research is required to strengthen the results of this clinical observation study.

## Conclusion

The present study demonstrated significant improvement in radiologic and clinical outcomes in both the HP and HM groups. However, concomitant CC reconstruction with hook plate fixation demonstrated the less acromial osteolysis and the statistical differences in reduction maintenance with less clinical significance than hook plate fixation alone in acute high-grade ACJ dislocations.

## Data Availability

The datasets analyzed during the current study are available from the corresponding author on reasonable request.

## Abbreviations

ACJ: Acromioclavicular joint
CMS: Constant Murley Score
UCLA: The University of California at Los Angeles
CCD: Coracoclavicular distance
ACD: Acromiocoracoid distance
aCCD: Absolute coracoclavicular distance
rCCD: Relative coracoclavicular distance
CC: Coracoclavicular
VAS: Visual analog scale
